# An empirical study of leadership, organizational culture, conflict, and work ethic in determining work performance in Indonesia's education authority

**DOI:** 10.1016/j.heliyon.2021.e07698

**Published:** 2021-07-30

**Authors:** Kiki Farida Ferine, Reza Aditia, Muhammad Fitri Rahmadana, Indri

**Affiliations:** a Postgraduate School, Universitas Pembangunan Panca Budi, Indonesia; b Postgraduate School, Universitas Negeri Medan, Indonesia; c Faculty of Economics, Universitas Negeri Medan, Indonesia

**Keywords:** Conflict, Leadership, Organizational culture, Work ethic, PLS-SEM

## Abstract

This study aimed to examine the influence of conflict, leadership, organizational culture, and work ethic on employees' work performance in North Sumatra Education Authority, Indonesia. This becomes important because this organization is not a profit-oriented organization, so it needs further understanding about how to foster the work performance. However, most of these research topics still concentrate on the western populations. A quantitative approach was used to conduct this study, where data were collected directly to the office of Education Authority with n = 180. Partial Least Square Structural Equation Modeling (PLS-SEM) is employed for data analysis in this study. The results showed that conflict negatively affects employees' work performance. However, leadership, organizational culture, and work ethic have positive effect on employees' work performance.

## 1 Introduction

In today's work environment, efforts to improve employee performance are almost the primary goal of human resources (HR). HR needs to be managed professionally to create harmony between the interests of employees and the interests of the organization in an effort to advance the organization (Mappamiring et al., 2020). Moreover, this is the role of a leader, because a leader's role in an organization is very dominant (Bauer et al., 2006; Hall et al., 2001; Salisbury, 1984; Schein, 1983), also the essence of leadership in an organization is to influence and facilitate individual and collective efforts to accomplish their objectives (Yukl, 2012). Leadership is known as an essential factor that determines the high and low of employee work performance in an organization (Al Khajeh, 2018; Berson et al., 2008; McColl-Kennedy and Anderson, 2002; Raja et al., 2020; Sonmez Cakir and Adiguzel, 2020). However, the leadership factor alone is known to be insufficient in maximizing employee performance. Several predictor variables are also suspected to affect work performance, namely organizational culture, conflict, work ethics, and work performance (Barker et al., 1987; Graham et al., 2017; Lau and Cobb, 2010; Lee et al., 2011; McColl-Kennedy and Anderson, 2002; O'Reilly, 1989; Schaubroeck et al., 2011; Wang et al., 2014).

Organizational culture is a set of norms or values widely applied to an organization (Guiso et al., 2015; O'Reilly et al., 2014). How organizational culture in an organization cannot be underestimated, is because organizational culture plays a role in giving identity to an organization (Cheung et al., 2011). Crémer (1993) states that organizational culture is the unspoken code of communication among members of an organization. Graham et al. (2017) reported that as many as 91% of executives view culture as something fundamental in their company, and 78% view culture as one of the top 3 factors that impact their company's value. Thus, culture can act as a “social control.” This is because each individual cares about the people around him (O'Reilly, 1989). Furthermore, as mentioned by Crémer (1993) it is assumed that human beings are honest and trustworthy, however they have limited capacity for processing, receiving, and transmitting information. It makes culture is defined as the stock of knowledge shared by the members in a particular organization. The acquisition of this knowledge is an investment.

Some previous research has also revealed that work conflicts also receive attention regarding the smooth running of an organization's journey (Lau and Cobb, 2010). Because conflict and the world of organization are actually two things that cannot be separated, even Tjosvold (2008) states that “to work in an organization is to be in conflict”. Indeed, it is known that conflict has several benefits to organizational climates, such as preventing premature agreement (Stasser and Birchmeier, 2003). In addition, in certain situations, conflict can also increase the creativity of its employees (De Clercq et al., 2017). However, if too many conflicts occur, instead of positively impacting the organization, it will become an obstacle to the organization. Various studies have shown that conflict has a high correlation with bullying behavior in organizations (Ayoko et al., 2003), harsh personality, and aggressive behavior (de Vliert, 1998). If this is not managed correctly, it will result in high turnover in the organization. Various studies examining the effects of conflict in different fields of work have proven this effect (Blomme et al., 2010; de Clercq et al., 2009; Sharma and Nambudiri, 2015). Conflict can be interpreted as a disagreement over interest or idea in an organization. However, generally, individual conflicts usually occur when someone has uncertainty about what tasks to do, which is due to the supervisor's unclearness (Henry, 2009). Conflict can be responded to in two different approaches. Destructive reaction to conflict is when the parties involved choose to avoidance, or each party tries hard to win the fight (Barker et al., 1988). The second approach is productive conflict. A productive conflict is a constructive approach to conflict that occurs as people cope with their incompatible activities and then try to solve their conflict (Tjosvold, 1985). Indeed, conflicts are rarely resolved quickly, but conflicts must still be appropriately managed so that the company or organization can move forward (Barker et al., 1987).

Work ethic has also been shown to influence performance (Blau and Ryan, 1997; Meriac, 2015). This relationship between effort-performance appears not only in the context of work but also in academic/educational pursuits (Meriac et al., 2015). The emergence of this concept originated from the work of Weber (1958). However, the work ethic discussed by Weber (1958) has a Protestant work ethic context. Over time, these paradigm shifts, from religious perspectives on work to the secularization of work (McCortney and Engels, 2003). When referring to studies discussing work ethic proposed by Weber, some of the behaviors associated with a strong work ethic are asceticism, integrity, independence, diligence, motivation, loyalty, and dependability (Hill, 1996; Kern, 1998). Furthermore, according to Miller et al. (2002), the developer of the Multidimensional Work Ethic Profile (MWEP), an inventory that is widely used to measure the construction of work ethic, seven dimensions form the work ethic, namely: work centrality, independence, hard work, comfort, morality/ethics, Gratification Delay, and Waste of Time. In general, work ethic is defined as a set of beliefs and attitudes that reflect the fundamental values of work (Meriac et al., 2010). Besides, work ethic also plays a role as a personality construct (Merrens and Garrett, 1975; Mirels and Garrett, 1971) and tends to remain unchanged (stable) from time to time (Ter Bogt et al., 2005).

Therefore, this study aims to capture a broader set of related to work performance, especially in Indonesia's education authorities employees. This becomes important because this organization is not profit-oriented, so it needs further understanding. As far as the researchers know, most of these research topics still concentrate on the western populations. In contrast, in Indonesia itself, the research discusses how conflict, leadership, organizational culture, and work ethics in shaping work performance in an organization have not yet been studied. Thus, we are hoping we can better understand the eastern population. Hence, the following hypotheses are proposed:

H1 Conflict is negatively related to work performance

H2 Leadership is positively related to work performance

H3 Organizational Culture is positively related to work performance

H4 Work Ethic is positively related to work performance

## 2 Materials and methods

### 2.1 Measurements

**Figure 1:**
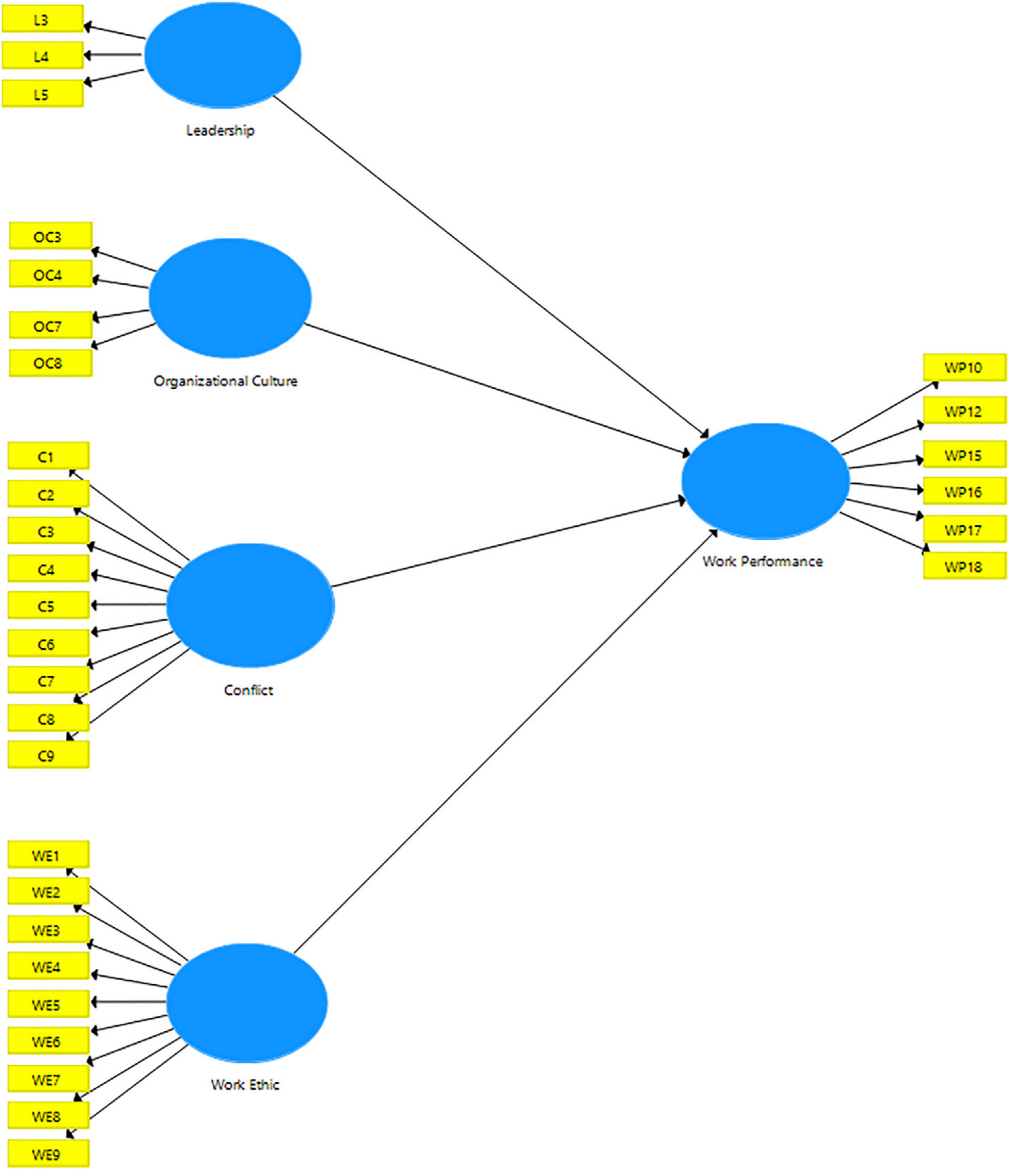
Research model.

Fifty-four items were generated to reflect the five constructs. The response format was a 5-point, likert type scale utilizing very agree to very disagree as end points. However, at the end, thirty-one were used to measure each construct because the rest have inadequate factor loading and AVE (see Figure 1).

### 2.2 Population and sample size

**Figure 2:**
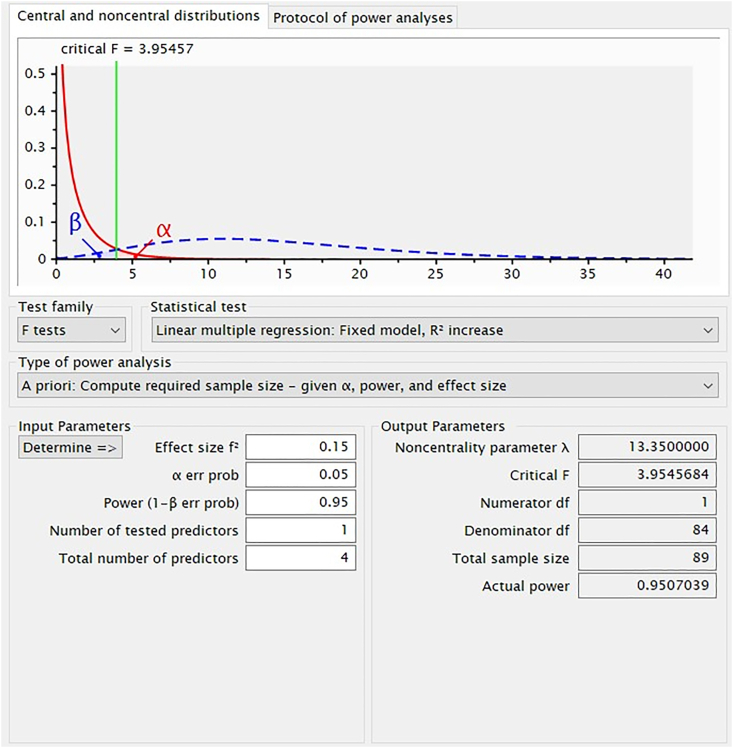
Power results for required sample size.

The population in this study were all employees who worked at the North Sumatra Province Education Autoritiy, Indonesia, totaling 536 people. Several can be used as a benchmark in taking the number of samples for SEM-PLS statistical analysis. Referring to Barclay et al. (1995), the sample size is at least ten times of the indicators used to measure the construct. Nevertheless, this basis was still considered too harsh. Thus the authors refer to the recommendation by Hair et al. (2016) who recommend that the sample size be adjusted according to power analysis. That is why to determine the number of samples that are suitable for power analysis, the author uses the help of G ∗ power software (Faul et al., 2007). We use error measurements of type one and two at α = 0.05 and β = 0.95, while the effect size = 0.15, and the number of predictors as the model offered by the researcher is 4. The settings author used to analyze the sample size and the results provided by the G ∗ powe application can be seen in Figure 2.

Figure 2 shows that at an error probability of 0.05 and a confidence level of 95%, the minimum sample required is 89 samples. This shows that the number of samples in this study is more than sufficient because the sample in this study uses a sample size of 190 samples.

### 2.3 Data collection

Data collection using a questionnaire survey distributed directly to the office of Education Authority in North Sumatra Province, Indonesia, a total of 180 respondents' answers (all samples) were collected. With a total sample (n = 180) divided into 113 men (62.78%) and 67 women (37.22%). Meanwhile, when the samples viewed from the level of education, the sample is divided into 16 samples of high school graduates (8.89%), 36 samples of diploma (20%), 101 samples (56.11%) of bachelor, 17 samples of masters (9.44%). and Ph.D. as many as 10 samples (5.56%) (see Table 1).

**Table 1:** Description of the respondents' characteristics.

		Count	Percentage
Gender	Male	113	62.78
	Female	67	67.22
Education	High School	16	8.89
	Diploma	36	20
	Bachelor	101	56.11
	Masters	17	9.44
	PhD	10	5.56

In collecting the data, ethical approval was granted by Universitas Pembangunan Panca Budi ethical committee, and consent was obtained from all participants in this study.

### 2.4 Data analysis

Partial Least Square Structural Equation Modeling (PLS-SEM) is employed for analyzing the data in this study. Although covariance-based structural equation modeling (CB-SEM) has dominated previous research as a method for analyzing complex interrelationships between observed and latent variables, in recent years, studies using PLS-SEM have increased much more rapidly than those using CB-SEM (Hair et al., 2016). In fact, PLS-SEM has now been widely applied in many social science disciplines, including in the fields of management (Ali et al., 2018; Hair et al., 2012, 2019; Kaufmann and Gaeckler, 2015; Peng and Lai, 2012; Ringle et al., 2012; Sinkovics et al., 2016; Sosik et al., 2009). In addition, the PLS-SEM analysis method is also desirable to many researchers because it allows them to estimate complex models with many constructs, indicators, and structural paths without having to force distributional assumptions on the data (Hair et al., 2019).

Two main steps were performed in analyzing the output results on Smart PLS v. 3.2.9, namely evaluation of measurement models and evaluation of the structural model (Hair et al., 2016; Ringle et al., 2015). Explanations for both evaluation will be explained in the next session.

## 3 Results

### 3.1 Evaluation of measurement models

The first step is examining the measurement model. Measurement model evaluation measures the reliability and validity of the constructs with their corresponding items. There are three aspects in determining the acceptance of the measurement model, namely convergent validity, internal consistency reliability, and discriminant validity. Referring to Hair et al. (2016), convergent validity is the degree to which a measure correlates positively with alternative measures of the same construct, required loading factors to exceed 0.5, while Average Variance Extracted (AVE) to exceed 0.5. Moreover, internal consistency reliability is a form of reliability used to judge the consistency of results across items on the same test, and determines whether the items measuring a construct are similar in their scores, it requires composite reliability >0.6, as well as the Cronbach's Alpha. The last aspect is discriminant validity, it is the extent to which a construct is truly distinct from other constructs by empirical standards. The cross-loadings and Fornell-Larcker criterion are typically used to assessing discriminant validity. Nevertheless, current research that critically examined the performance of cross-loadings and the Fornell-Larcker criterion for discriminant validity has found that neither procedure reliably recognizes discriminant validity issues (Henseler et al., 2015). As a remedy, Henseler et al. (2015) have suggested to use Heterotrait-monotrait ratio (HTMT). For the threshold level, Heterotrait-Monotrait ratio (HTMT) confidence interval must not include 1, while a lower and thus more conservative threshold value of 0.85 seems warranted (Henseler et al., 2015).

In the Smart PLS analysis, the authors used a bootstrapping of 5000 sub-samples as recommended by Hair et al. (2016). In the first analysis, the measurement model does not meet the requirements because it has a low AVE value, so there are several indicators with low loading factors that are removed, namely L1, L2, L6, L7, L8, L9, OC1, OC2, OC5, OC6, OC9, WP1, WP2, WP3, WP4, WP5, WP6, WP7, WP8, WP9, WP11, WP13, and WP14. After the new model is formed, we run the PLS algorithm for the second time. As we can see in Table 2, the results demonstrated that all constructs present adequate convergent validity, with loadings and AVE exceed 0.5. Internal consistency reliability also exceeded the threshold, with composite reliability and Cronbach's alpha exceeding 0.6. With regard to discriminant validity (Table 3), HTMT was applied, and the measurement results showed that there is no single construct that includes 0.85 in HTMT.

**Table 2:** Results summary for convergent validity and internal consistency reliability.

Latent Variable	Indicators	Convergent Validity					Internal Consistency Reliability			
		Standard Deviations	Mean	Loadings	AVE	Sig. Level	Standard Deviations	Mean	Composite Reliability	Cronbach's Alpha
Leadership	L3	0.05	0.91	0.92	0.80	0.00	0.04	0.92	0.921	0.872
	L4	0.05	0.93	0.93		0.00				
	L5	0.07	0.82	0.83		0.00				
Organizational Culture	OC3	0.04	0.74	0.74	0.53	0.00	0.02	0.82	0.819	0.706
	OC4	0.05	0.72	0.72		0.00				
	OC7	0.04	0.77	0.77		0.00				
	OC8	0.06	0.68	0.69		0.00				
Conflict	C1	0.16	0.63	0.68	0.54	0.00	0.12	0.88	0.915	0.899
	C2	0.19	0.69	0.75		0.00				
	C3	0.15	0.74	0.79		0.00				
	C4	0.15	0.75	0.81		0.00				
	C5	0.15	0.70	0.75		0.00				
	C6	0.14	0.70	0.75		0.00				
	C7	0.15	0.65	0.70		0.00				
	C8	0.15	0.62	0.68		0.00				
	C9	0.15	0.70	0.73		0.00				
Work Ethic	WE1	0.05	0.67	0.68	0.51	0.00	0.01	0.90	0.901	0.876
	WE2	0.06	0.64	0.64		0.00				
	WE3	0.06	0.57	0.58		0.00				
	WE4	0.05	0.73	0.74		0.00				
	WE5	0.03	0.82	0.82		0.00				
	WE6	0.04	0.74	0.75		0.00				
	WE7	0.04	0.77	0.77		0.00				
	WE8	0.04	0.74	0.74		0.00				
	WE9	0.06	0.66	0.66		0.00				
Work Performance	WP10	0.04	0.73	0.73	0.50	0.00	0.02	0.85	0.854	0.795
	WP12	0.06	0.64	0.65		0.00				
	WP15	0.05	0.70	0.71		0.00				
	WP16	0.08	0.62	0.62		0.00				
	WP17	0.04	0.78	0.78		0.00				
	WP18	0.05	0.73	0.73		0.00

**Table 3:** Result for discriminant validity – HTMT.

	Leadership	Conflict	Organizational Culture	Work Ethic	Work Performance
Leadership	---				
Conflict	0.169	---			
Organizational Culture	0.258	0.110	---		
Work Ethic	0.107	0.200	0.244	---	
Work Performance	0.281	0.163	0.834	0.428	---

### 3.2 Evaluation of structural model

After the construct measures are confirmed to reliable and valid, the next step is to make the assessment of the structural model results. According to Hair et al. (2016), when examining the structural model, it is important to understand that PLS-SEM is different from CB-SEM, which estimates parameters so that the differences between the sample covariances and those predicted by the theoritical/conceptual model are minimized. The goodness-of-fit measures such as the chi-square statistic or the various fit indices associated with CB-SEM not fully transferrable to PLS-SEM. Instead, the key criteria for assessing the structural model in PLS-SEM are the path coefficients, *R*^2^ values, *f* ^2^ effect size and SRMR.

Structural model evaluation is to test the path among constructs based on the stated hypothesis. As suggested by Hair et al. (2016), we used bootstrapping with 5000 subsamples, two-tailed, and 0.05 significant level to produce the standard error and t-statistics for the sample. As shown in Table 4, the structural model assessment results revealed that the four main paths are significant. Table 4 also shows that the path relationship between conflict and work performance is significant β = - 0.132, p = 0.05. This indicates that conflict has a negative significant effect on work performance. On the other hand, leadership shows that there is positive significant effect on work performance, β = 0.126, p = 0.027. Organizational culture also showed positive significant effect on work performance, with β = 0.562, p = 0.00. In addition, work ethic showed positive significant effect on work performance as well, β = 0.219, p = 0.000. It means that unlike conflict; leadership, organizational culture, and work ethic have positive effect on work performance.

**Table 4:** Results summary for structural model evaluation.

	Coefficient	Mean	Standard Deviation	t values	P values
Path Coefficient					
Conflict -> Work Performance	-0.132	-0.151	0.067	1.961	0.050
Leadership -> Work Performance	0.126	0.130	0.057	2.211	0.027
Organizational Culture -> Work Performance	0.562	0.559	0.052	10.737	0.000
Work Ethic -> Work Performance	0.219	0.222	0.052	4.194	0.000
r square	0.482	0.510	0.055	8.768	0.000
f square					
Conflict -> Work Performance	0.032	0.053	0.031	1.029	0.304
Leadership -> Work Performance	0.029	0.038	0.030	0.958	0.338
Organizational Culture -> Work Performance	0.564	0.597	0.159	3.548	0.000
Work Ethic -> Work Performance	0.086	0.097	0.046	1.872	0.061
SRMR	0.063	0.062	-	-	-

Next, the most commonly used measure in evaluating the structural model is the coefficient of determination (*R*^2^ value). The coefficient represents the amount of variance in the endogenous constructs explained by all of the exogenous constructs linked to it (Hair et al., 2016). The value ranges from 0 to 1. While it is challenging to present rules of thumb for adequate *R*^2^, however, 0.20 are considered adequate (Hair et al., 2016). As we can see from Table 3, the *R*^2^ coefficient is 0.482, so it means the *R*^2^ is adequate and this implies that the four exogenous constructs explain 48.2% of the variance of endogenous construct.

**Figure 3:**
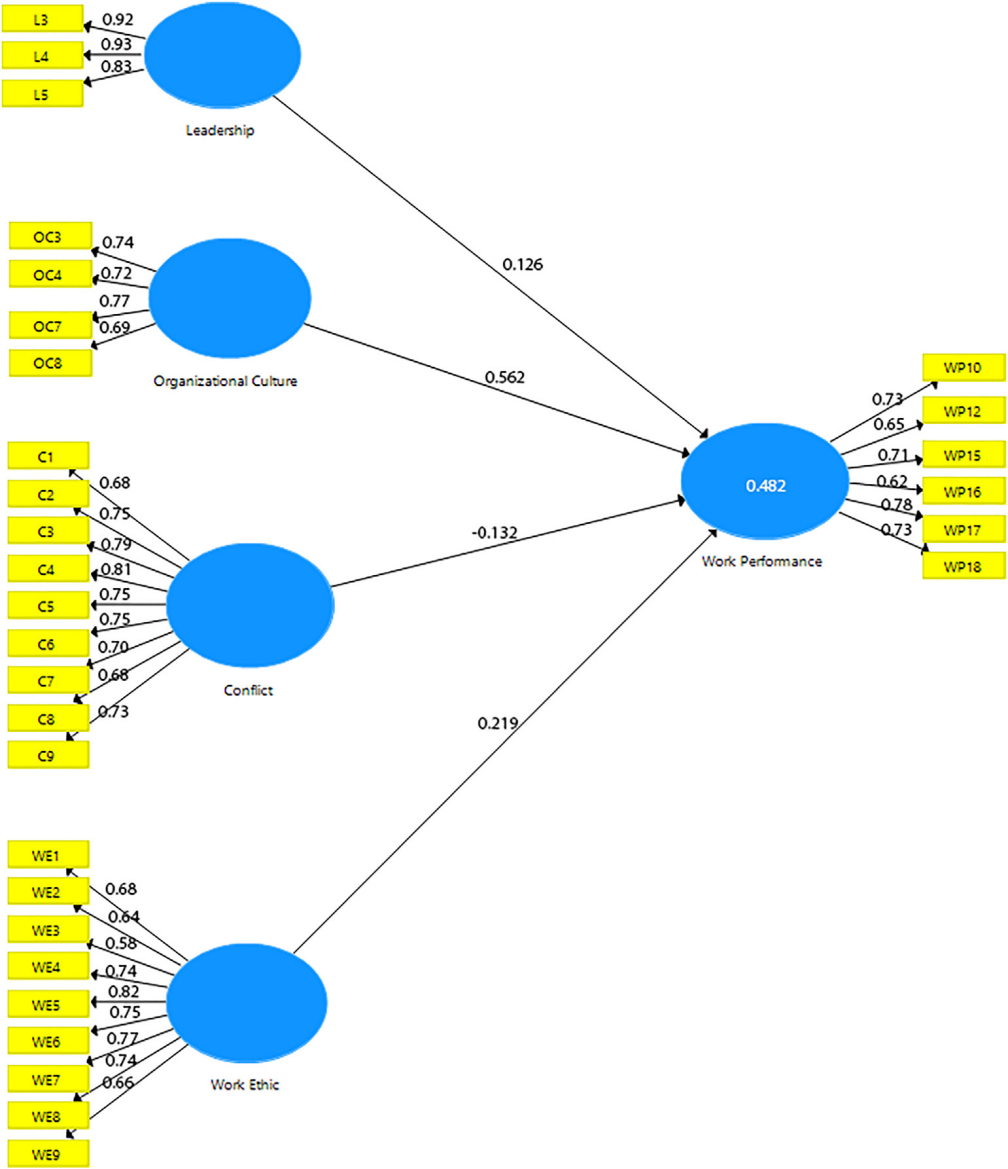
Structural Model with loading factor, path coefficients, and r square.

Furthermore, the effect size of the predictor constructs were evaluated using ƒ^2^ effect size. Guidelines for evaluating ƒ^2^ are that values of 0.02, 0.15, and 0.35, sequentially represent small, medium, and large effects (Cohen, 2013). In consequence, from Table 4 we can concluded if Conflict and Leadership considered as medium effect size, wile Organizational Culture and Work Ethic were considered as large effect size. SRMR also assessed to know the root mean square discrepancy between the observed correlations and the model-implied correlations (Hair et al., 2016). Because the SRMR is an abolute measure of fit, a value of zero indicates perfect fit. However, following a conservative approach, an SRMR value of less than 0.08 indicates good fit. From Table 4, as we can see SRMR has value of 0.063. Hence, the SRMR indicates good fit of the model. In term of results of latent variable correlation, can be seen in Table 5 (see Figure 3).

**Table 5:** Results of latent variable correlations.

	Coefficient	Mean	Standard Deviation	T-value	P Values
Leadership -> Conflict	0.143	0.118	0.074	1.942	0.052
Organizational Culture -> Conflict	0.010	-0.019	0.074	0.138	0.890
Organizational Culture -> Leadership	0.203	0.208	0.077	2.615	0.009
Work Ethic -> Conflict	-0.178	-0.184	0.079	2.243	0.025
Work Ethic -> Leadership	0.075	0.082	0.073	1.021	0.308
Work Ethic -> Organizational Culture	0.194	0.204	0.073	2.662	0.008
Work Performance -> Conflict	-0.147	-0.187	0.081	1.813	0.070
Work Performance -> Leadership	0.238	0.246	0.078	3.050	0.002
Work Performance -> Organizational Culture	0.629	0.634	0.051	12.443	0.000
Work Performance -> Work Ethic	0.361	0.376	0.061	5.967	0.000

## 4 Discussion

This study examined the effect of conflict, leadership, organizational culture, and work ethic on employees' work performance. Therefore, we use SEM-PLS to analyze the data. The results support the reliability and validity of the measurement model (Table 2 and Table 3).

From the structural model evaluation, it was first observed that the *R*^2^ coefficient is 0.482, which is adequate. Concerning hypothesis testing, the empirical results for the samples showed that conflict has negative effect on employeess's work performance. Hence, this result complies with Lau and Cobb (2010), who found that conflict can negatively affect employees' work performance. Besides, the results are also compatible with prior studies that proved the negative impact of conflict on employees' work performance (Jehn and Bendersky, 2003; Pelled et al., 1999). Pelled et al. (1999) even found that diversity sometimes shapes conflict and that conflict, in turn, shapes performance. However, these linkages are subtleties. According to affective event theory, negative emotions influence individuals' attitudes and behaviors more than positive emotions (Weiss and Cropanzano, 1996). Not only that, a study by Rispens and Demerouti (2016) also found that conflict event not only increases anger and contempt but guilt and sadness as well. However, the findings of research conducted by De Clercq et al. (2017) prove otherwise. They found that task conflict positively affected employees, as it was found that task conflict could increase employee creativity. Nevertheless, this positive impact has requirements; task conflict can only enrich creativity only for employees who have higher levels of learning orientation. If it is known that employees in an organization do not have a higher level learning orientation, it is better to keep conflicts in the work environment to a minimum level. This is where the role of leaders becomes essential in carrying out conflict management behavior, to overcome conflict-stress relationships of employees (Römer et al., 2012).

In addition, leadership was discovered to be positively and significantly affect the work performance of employees. It seems logical that leadership in organizations can influence and facilitating individual and collective efforts to accomplish shared objectives (Yukl, 2012). The result is congruent with prior empirical research that proved the positive effect of leadership on work performance (Rus et al., 2010; Wang et al., 2014). Leadership is very important because it influences employee behavior by gradually changing their values corresponding closer to those of the learning organization (Ribière and Sitar, 2003), and when employees perceive top managers as trustworthy, a firm's performance is stronger. However, the literature that discusses in more detail what leadership style can shape employees' work performance also needs to be considered. This is because, referring to the results of research conducted by other scholars, not all leadership style can foster work performance. This is due to the leadership style that affects work performance is transformational leadership (Dvir et al., 2002; Erkutlu, 2008; Thamrin, 2012; Walumbwa et al., 2008). This topic is a limitation in this study because this study does not divide the leadership style more specifically. Furthermore, the authors would like to contradict the research findings conducted by Chen and Silverthorne (2005) and Paais and Pattiruhu (2020), which stated no relationship between leadership and employee job performance. This finding contrasts with the authors' findings, who found that leadership positively and significantly influenced work performance. Moreover, authors' finding is also supported by many other scholars (Ribière and Sitar, 2003; Rus et al., 2010; Wang et al., 2014; Yukl, 2012). Differences in research results may be based on Chen and Silverthorne (2005) who use statistical techniques that are not suitable. Even in the article, they do not explicitly explain what statistical analysis had been used.

Moreover, the PLS results also explained that organizational culture has a significant positive influence on employees' work performance. Although this fact sounds reasonable and doubtless, empirical evidence is somewhat thin (Berson et al., 2008; Peterson et al., 2003). Graham et al. (2017) mentioned that cultural norms are as important as stated values in achieving success. That is why this study enriches the finding from the previous study. This study's results are consistent with prior studies that have asserted that corporate culture promotion affects performance in terms of innovation output (Zhao et al., 2018). In addition, 91% of executives believe culture is important to their firms, and 79% place culture among the top 3 or the top 5 value drivers (Graham et al., 2017). This is also following earlier literature that indicated if organizational culture as crucial role in employees' work performance (Alvesson, 2012; Ouchi and Wilkins, 1985; Schein, 1990). In terms of enriching the findings of research conducted by previous scholars, the authors also wish to refute the research findings conducted by Pawirosumarto et al. (2017), which states that organizational culture does not significantly and positively influence employees' performance. The authors also doubts the research findings conducted by Pawirosumarto et al. (2017) because they do not explain the assumption test before carrying out statistical analysis. Whereas as is known, CB-SEM is a parametric test that requires the data to meet the assumption, such as multivariate normality (Hair et al., 2014, 2017).

The study's findings also showed that work ethic was found to be positive and significantly influence employees' work performance. Moreover, these results support the argument if work ethic significantly affects performance, both directly and indirectly through innovative work behavior (Javed et al., 2017). This because work ethic comprises an individual's ethical behavior, so they tend to work wholeheartedly (Khan et al., 2013). Individuals who have strong ethical behavior, emphasize hard work with a high level of devotion to meet the task request requirement by their organization (Schneider, 1990). Apart from being a predictor, work ethics also acts as a mediator in influencing employees' work performance in an organization. Referring to the research results conducted by Raja et al. (2020), despotic leadership was able to affect job performance significantly when Islamic Work Ethic was high. With the role of the work ethic, either as a predictor or a mediator variable, the supervisor's attention to the work ethic that employees have in their organization is essential. Do not let the decline in work ethics happen to employees in an organization because its impact on performance is significant.

## 5 Conclusion

The emerging of the work environment makes organizations need to transform how they run their organization. Numerous frameworks have been presented in recent years. Thus, understanding how to achieve optimal work performance is crucial. Hence, this study proposes a framework to achieve it. Five factors, namely leadership, organizational culture, conflict, and work ethic, were hypothesized to determine employees' work performance.

The proposed model effectively explains the constructs of work performance with R^2^ = 0.482. From the evaluation of the structural model, all the proposed hypotheses are found to be positively and significantly influence the work performance except conflict, which found to have a negative and significant effect on work performance. This finding suggests that to attain stellar work performance, the organization needs to foster supportive leadership. At least when referring to Yukl (2012), there are several specific behaviors that an effective leader should have, namely.

1. Task-Oriented Behaviors, including the ability to plan, clarifying, monitoring, and problem-solving,
2. Relations-Oriented Behaviors, including the ability to support, develop, recognize, and empower,
3. Change-Oriented Behaviors, including the ability to advocate change, envisioning change, encouraging innovation, and facilitating collecting learning,
4. External Leadership Behaviors, including networking skills, external monitoring, and representing.

In addition, this study also suggests that organizations pay more attention when recruiting people at the executive level. This is because a leader's personality (introvert or extrovert) also affects employees' work performance (Bauer et al., 2006). Ideally, an organization should reduce the turnover of people at the executive level because, besides the expensive recruitment process, some organizations must keep their company secrets. Several ways can be done, such as providing tests that measure personality types, as well as leadership measuring instruments, e.g., empowering leadership questionnaire (ELQ) (Arnold et al., 2000). However, in terms of organizational culture, this factor has a significant positive effect on employees' work performance. This finding suggests that organizations engage in activities that build a constructive organizational culture. For example, Pixar always reflects on the films they made and is not reluctant to build a constructive criticism culture (Catmull and Wallace, 2014). Of course, this cannot be replicated entirely, because nevertheless, organizations need to find their own culture to build on. The role of leaders in shaping organizational culture is also very influential because CEOs who have openness to new experiences tend to create an organizational culture where they also tend to have high adaptability (O'Reilly et al., 2014). Furthermore, even this continue to adapt culture has a good influence on organizational success, and it is not surprising that companies that have a continue to adapt culture tend to be able to book high profits for the company (O'Reilly et al., 2014). Results also showed that conflict has negative effect on work performance. This result, of course, is related to the spread of conflict in the work environment, making communication between employees disrupted. This research suggests that leaders resolve misunderstandings between employees as early as possible. The communication disruption between conflicting employees will also damage the discussion or meeting process in the organization, which impacts employee performance. Finally, this study has shown that work ethic positively and significantly influences work performance. This implies that it is essential to ensure the recruited people have a high work ethic and create a supportive atmosphere for employees to continue to be honest in their daily work. The implication of this is that company leaders can see the level of religiosity of employees or prospective employees because someone who has a high level of religiosity tends to have a high work ethic (Javed et al., 2017; Raja et al., 2020; Weber, 1958). This situation is not surprising because the concept of work ethics itself was originally based on the concept of theology (Weber, 1958). Regularly measuring employees' work ethics with inventory that has been popularly used, such as MWEP (Meriac et al., 2013), can also be used. This is intended as a preventive measure for the decline in employee performance in an organization. After the organization finds employees suspected of having a low level of work ethics, the organization can provide counseling to improve their work ethic.

## Declarations

### Author contribution statement

Kiki Farida Ferine: Conceived and designed the experiments; Performed the experiments; Wrote the paper.

Reza Aditia and Muhammad Fitri Rahmadana: Analyzed and interpreted the data; Wrote the paper.

Indri: Contributed reagents, materials, analysis tools or data; Wrote the paper.

### Funding statement

This research did not receive any specific grant from funding agencies in the public, commercial, or not-for-profit sectors.

### Data availability statement

Data will be made available on request.

### Declaration of interests statement

The authors declare no conflict of interest.

### Additional information

No additional information is available for this paper.
